# Physicochemical Properties, Fatty Acid Composition, and the Effect of Heating on the Reduction of Cyclopropenoid Fatty Acids on Baobab (*Adansonia digitata* L.) Crude Seed Oil

**DOI:** 10.1155/2020/6691298

**Published:** 2020-12-14

**Authors:** Upendo L. Msalilwa, Edna E. Makule, Linus K. Munishi, Patrick A. Ndakidemi

**Affiliations:** ^1^Department of Sustainable Agriculture, Biodiversity and Ecosystem Management, The Nelson Mandela African Institution of Science and Technology, Arusha, Tanzania; ^2^Centre for Research, Agricultural Advancement, Teaching Excellence and Sustainability (CREATES) in Food and Nutrition Security, The Nelson Mandela African Institution of Science and Technology, Arusha, Tanzania; ^3^Department of Food and Nutritional Sciences, The Nelson Mandela African Institution of Science and Technology, Arusha, Tanzania

## Abstract

The baobab seed oil has been consumed by humans due to its medicinal and nutrient values for many years. However, the consumption of baobab seed oil has been perceived by different communities as a health risk caused by cyclopropenoid fatty acids (CPFAs), which are carcinogenic ingredients present in the oil. This study investigated the physicochemical properties and fatty acid profile of baobab crude seed oil collected from semiarid areas in Tanzania and determined the effects of heating on the reduction of CPFAs. The baobab seed crude oil was extracted by Soxhlet using n-hexane, and the fatty acid composition of the baobab seed crude oil was determined by gas-liquid chromatography (GLC). Since CPFAs are resistant to lower temperatures, the effect of heating on the CPFA content of baobab crude seed oil was studied at 150°C, 200°C, and 250°C. The *A. digitata* crude seed oil was found to contain mainly twelve essential fatty acids and two different CPFAs. The most abundant fatty acids were palmitic acid, oleic acid, and linoleic acid in all the baobab population hotspots occurring in Tanzania. There was no significant difference in most physicochemical properties and fatty acid composition across the different semiarid areas in Tanzania. The major breakdown of CPFAs occurs at 200°C, and that would be the optimal temperature recommended for the refining process of the baobab crude oil. The study recommended refining of the baobab oil at higher temperatures ranging from 200 - 250°C as the best way of reducing CPFAs.

## 1. Introduction

The baobab (*Adansonia digitata* L.) is a tree species native to the semiarid areas of Africa, Australia, and Madagascar [1]. The tree is among the eight global species of baobab in the genus *Adansonia* from the family Malvaceae and subfamily Bombacaceae [2]. *A. digitata* is one of the most important tree species of nontimber forest products (NTFPs) that significantly contribute to the food and nutrition security of rural communities [3]. For example, pulp, leaves, and seeds of the tree are rich in food nutrients such as minerals, fatty acid, and vitamins [4–6]. The baobab leaves are rich in calcium ranging from 307 to 2640 mg/100 g dry weight (dw) and proteins with a chemical score of 0.81 [7]. The baobab pulp is rich in vitamin C; the consumption of 40 g of baobab pulp contains more than 80 percent of the Recommended Daily Intake (RDI) of vitamin C [7], which is suitable for pregnant women. The baobab seeds and its kernels contain high lipid content ranging from11.6 to 33.3 g/100 g dw and 18.9 to 34.7 g/100 g dw, respectively [8]. The pulp and leaves exhibit antioxidant properties with higher activity in the pulp than in the leaves [7].

The Malvaceae family has seed oil that contains essential fatty acids including palmitic, oleic, linolenic, and cyclic fatty acids (cyclopropene fatty acids) [9]. It has been reported that cyclopropene fatty acids are often accompanied by a smaller proportion of cyclopropanic fatty acids such as dihydrosterculic and dihydromalvalic acids, which are the dihydro analogues of cyclopropene fatty acids [10]. Studies revealed that CPFAs occur in lipids of the plant species including the seeds of baobab of the order Malvaceae, Tiliaceae, Bombacaceae, and Sterculiaceae families [11–13]. The CPFAs, which have been reported as frequent and dominant in baobab seed oil include sterculic acid [8-(2-octyl-1-cyclopropenyl) octanoic acid] and malvalic acid [7-(2-octyl-1-cyclopropenyl) heptanoic acid] [14–21].

The consumption of baobab seed oil has been reported to cause potential health risks caused by the presence of these carcinogenic ingredients [22–25] with medical and mutagenic effects on animals [26, 27] and carcinogenic effects [28, 29]. Despite the increased consumption and popularity of baobab oil due to its medicinal values, FDA [30] issued an alarming statement condemning the consumption of baobab oil for health reasons. Furthermore, according to [30], the health effect of baobab is caused by the presence of carcinogenic ingredients known as CPFAs. Although the consumption of baobab oil is discouraged in Tanzania, evidence shows that most people are still using it for the treatment of various diseases.

In sub-Saharan Africa, baobab seed oil has been used for many years by local populations for medicine, beauty, and food purposes [31]. In Tanzania, local people use baobab seed oil for medicinal purposes. Several ethno pharmacological studies have reported that baobab seed oil possesses several biological activities such as antioxidant, prebiotic, anti-inflammatory, analgesic, antipyretic, antidiarrhoea, antidysentery, and excipient [32]. Furthermore, baobab seed oil is effective against different conditions including hypertension, diabetes, obesity, and abdominal ailments [7]. These properties have made baobab products widely used in both traditional and modern medicines. However, the CPFAs in baobab oil ranged from 10 to 12.8 percent, which is far above the recommended level of 0.4 percent, which is suitable for human consumption [30]. Experiments on rats have shown stunted growth rate, an increase of the liver size, a delay in sexual development in females, alteration of fatty acid metabolism [29], and an increase in liver cancer incidences when fed in conjunction with aflatoxin *B1* or *M1* in rainbow trout [25, 29].

Furthermore, according to the World Health Organization (WHO) and the Food and Agriculture Organization (FAO), baobab oil is not recommended as edible oil, neither is it classified in the group of vegetable oils. Although the Government of Tanzania has banned the consumption of baobab oil, evidence shows that most people are still using baobab oil, which is known to have CPFAs, for the treatment of various diseases. The baobab oil requires postextraction treatments such as heat treatments in reducing or removing these cyclopropenoid fatty acids from the baobab seed oils before they are fit for human consumption. Accordingly, there is a need for characterizing and quantifying the levels of CPFAs in baobab seed oil and detecting the efficient method of removing/reducing their concentrations without impacting the quality of other fatty acids and oil quality. Therefore, this study characterized the essential fatty acids and cyclopropenoid fatty acids (CPFAs) present and determined the effect of heat on cyclopropenoid fatty acids in baobab seed crude oil in the semiarid areas of Tanzania.

## 2. Material and Methods

### 2.1. Study Area Description

**Figure 1:**
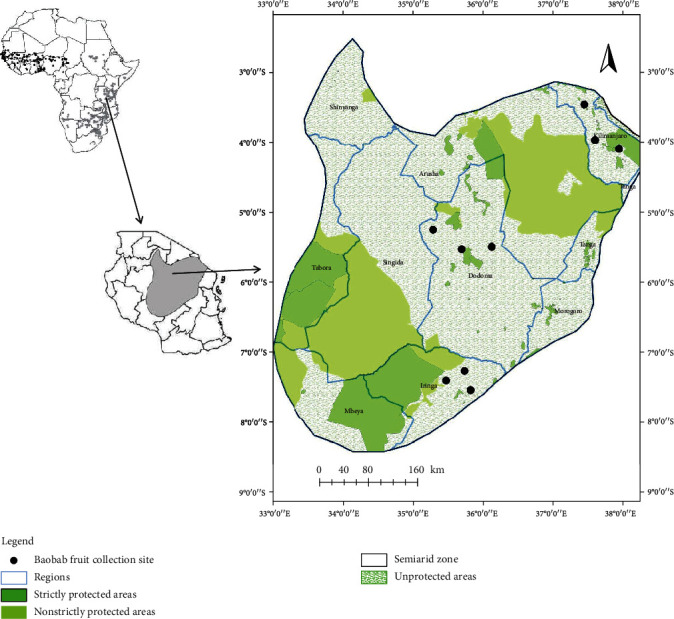
Map showing the location of the study area in Tanzania.

The study was conducted in the semiarid region in Tanzania located between latitude: 2°39′5.225^″^S, longitude: 34°8′29.364″E and latitude: 8°2′53.048^″^S, longitude: 35°3′18.731^″^E (Figure 1). The altitude of the area ranges from 490 m to 1400 m. The semiarid areas in Tanzania receive rainfall of less than 800 mm annually and are characterized by a low amount of rainfall, high evapotranspiration rates, and erratic temporal and spatial distribution of rainfall [33]. The mean monthly minimum and maximum temperatures are 26°C and 30°C, respectively. In this respect, these areas have inherently low and unreliable crop and livestock production [34]. The human population density of the semiarid areas is approximately 62 persons per square kilometre [35]. The main human activities practiced in the semiarid areas are mainly crop farming and grazing.

### 2.2. Baobab Fruit Collection

The baobab fruits were collected during an ecological survey in Dodoma, Iringa, and Kilimanjaro regions (Figure 1). The selection criteria for the regions were the presence, accessibility, and use of baobab trees and their products. Nine *A. digitata* trees (three baobab trees in each region) were randomly selected for the collection of mature fruits. About 20 baobab mature and intact fruits were harvested from each tree. These fruits were collected during the dry season (August 2018–February 2019). The geographical and environmental data were recorded in each sampled tree (Table 1).

### 2.3. Crude Oil Extraction and Heat Treatment

Hard woody shells were removed manually from baobab fruits from each area, and these were carefully crushed to separate the seeds from the pulp using a pulp seed separator machine. The obtained seeds were further subjected to cold pressing for oil extraction while ensuring that the initial crude oil for analysis was prevented from contamination. The extracted crude baobab seed oil was kept in an amber-light bottle covered with its glass cover and immediately taken to the laboratory for analysis.

### 2.4. Analysis of Baobab Crude Oil

#### 2.4.1. Determination of the Physicochemical Properties

The physicochemical properties of baobab crude oil were determined following the methods of [36]. The baobab crude oil was obtained from 20 mature and intact baobab fruits (harvested from 3 trees per region) in which the physicochemical property parameters were analysed in three replicates per region. These parameters include free fatty acid (mEq/100 g oil), specific gravity (25°C/25°C), refractive index (27°C), unsaponification value, saponification matter, peroxide value, and iodine value. A total of twenty-one (21) samples were analysed for the physicochemical properties of baobab crude oil which included three replicated per region.

*(1) Free Fatty Acids (FFA)*. Five (5) grams of homogenized and sieved crude oil sample was weighed in a 250 ml conical flask. About 50-100 ml of freshly neutralized hot ethyl alcohol was added to the oil sample followed by the addition of about 1 ml of phenolphthalein indicator. The mixture was boiled for about five minutes and titrated while hot, against standard 0.1 N potassium hydroxide solution while shaking vigorously until the mixture formed a pink colour, which persists for about 15 minutes. Free fatty acid was obtained by equation (1).

*(2) Specific Gravity (SG)*. Five (5) grams of baobab crude oil sample was warmed to 45°C to melt oil molecules. Then, the samples were left to cool until they reached a temperature of about 30°C. The dry pycnometer was filled with the prepared sample in such a manner that prevented entrapment of air bubbles after removing the cap of the sidearm. The stopper was inserted, immersed in a water bath at 30°C, and held for 30 minutes. The bottle was removed from the water bath, cleaned, dried thoroughly, and weighed ensuring that the temperature did not fall below 30°C. Specific gravity was calculated using equation (2).

*(3) Refractive Index (RI)*. The refractive index was measured at 25°C by a Pen Refractometer (Atago, Japan) with resolution and accuracy values of 0.1 and ±0.2 percent at 10-60°C. The refractometer was set at 583.9 nm and left warm for 30 minutes. The oil was poured on the double prism with the help of a screw head. The prisms were closed by tightening the screw heads. Then, the refractometer values were taken and translated using a conversion table.

*(4) Saponification Value (SV)*. About 2.0 g of crude oil sample was added into a 250 ml Erlenmeyer flask, and then, 25 ml of the alcoholic potassium hydroxide solution was added. A blank sample was then prepared by placing 2.0 g of distilled water. The sample and the blank flasks were boiled in the boiling water bath for 1 hour under a reflux condenser. After boiling, the flask and the condenser were cooled with about 10 ml of hot ethyl alcohol neutral to phenolphthalein. The excessive potassium hydroxide was titrated with 0.5 N hydrochloric acid using about 1.0 ml phenolphthalein indicator. The saponified matter was obtained using equation (3).

*(5) Unsaponified Matter*. Five (5) grams of well-mixed baobab crude oil sample was weighed into a 250 ml conical flask; then, 50 ml of alcoholic potassium hydroxide solution was added, and the content was gently boiled under a reflux air condenser for one hour. After boiling, the condenser was washed with 10 ml of ethyl alcohol; the mixture was cooled and transferred to the separating funnel. About 50 ml of petroleum ether was added to the mixture in the separating funnel, shaken vigorously, and layers were allowed to separate. The lower soap layer was transferred into another separating funnel. The combined ether extract was washed three times with 25 ml portions of aqueous alcohol and then washed with 25 ml portions of distilled water to ensure that the ether extract is free of alkali (washing is no longer alkaline to phenolphthalein). The ether solution was transferred into a 250 ml beaker, evaporated to about 5 ml, and transferred quantitatively using several portions of ether into a 50 ml Erlenmeyer flask previously dried and weighed. The residues were dissolved in 50 ml of warm ethanol which was neutralized to a phenolphthalein endpoint and titrated with 0.02 N NaOH. The unsaponified matter was calculated using equation (4).

*(6) Peroxide Value (PV)*. Five (5) grams of baobab crude oil sample was weighed into a 250 ml stoppered conical flask. Then, 30 ml of acetic acid-chloroform solvent mixture was added and swirled to dissolve. About 0.5 ml saturated potassium iodide solution was added and left to stand for 1 min in the dark with occasional shaking, and then, about 30 ml of water was added. The obtained mixture was slowly titrated against the liberated iodine of 0.1 N sodium thiosulphate solution and vigorously shaken until the yellow colour was almost gone. About 0.5 ml starch solution was added as an indicator and continued titration while shaking vigorously to release all I_2_ from the CHCl_3_ layer until the blue colour disappeared. If less than 0.5 ml of 0.1 N Na_2_S_2_O_3_ is used, the process is repeated using 0.01 N Na_2_S_2_O_3_. Peroxide value expressed as milliequivalent of peroxide oxygen per kg sample (mEq/kg) was obtained by using equation (5).

*(7) Iodine Value (IV)*. About 5 g of baobab crude oil sample was weighed into a 250 ml conical flask with a glass stopper, and thereafter, 25 ml of carbon tetrachloride was added to it. The contents were thoroughly mixed followed by addition of 25 ml of Wij's solution, and a glass stopper was placed on it. The mixture was left to stand for 30 minutes with occasional stirring. Simultaneously, a blank was prepared by adding of 5 g of distilled water instead of a sample. After standing for 30 minutes, 15 ml of saturated potassium iodide solution was added, and then, 100 ml of recently boiled and cooled water was added, rinsing in the stopper. The liberated iodine was titrated with 0.1 N sodium thiosulphate solution using starch as an indicator until the formed blue colour disappeared after thorough shaking with the stopper on. Iodine value was calculated by using equation (6).

#### 2.4.2. Quantification and Characterization of Fatty Acids and CPFA in Baobab Crude Oil

Triplicates of baobab crude oil samples from each region were subjected to quantification and characterization of fatty acids and CPFAs. Nine (9) baobab crude oil samples were analysed from the three regions. The fatty acid quantification was done using gas chromatography of methyl esters [37].

*(1) Preparation of Baobab Crude Oil Sample for FA Profiling Analysis*. The fatty acid (FA) profiles of the crude oils were determined as fatty acid methyl esters (FAME) by gas chromatography through a method whereby alkaline hydrolysis is combined with boron trifluoride (BF_3_) catalyzed desterification [38]. The baobab crude oil was methylated by putting 2 mg of the sample in a flask and refluxing with 5 ml of 95 percent of methanol-HCL for 1 hour. The methyl esters were extracted with three portions of hexane (5 ml) and then washed with distilled water (5 ml). The hexane layer was dried in a vacuum rotary evaporator, and the residue was redissolved in 1 ml of hexane. Then, 1 *μ*l was injected into the GC under split mode of 60°C (Shimadzu GC-2010 equipped with an autosampler) with a capillary column, Supelco Carbowax size 30 m × 0.53 mm, injection temperature of 240°C, and detection temperature of 260°C under a flame ionization detector. Fatty acid methyl esters were identified by comparing the retention with standard times and expressed as percentages of the total methyl esters.

*(2) Preparation of the Methyl Esters for Cyclopropenoid*. Methyl esters were prepared from oils by refluxing approximately 100 mg of the crude oils with 5 ml of a solution of 1 percent sodium methoxide (0.5 N) in methanol. After 20 minutes, the solution was cooled, flooded with 15 ml of distilled water, and the methyl esters extracted twice with 10 ml of petroleum ether (BP 30-60°C). The esters were dried over anhydrous sodium sulfate and evaporated in a gentle stream of nitrogen just to dryness.

*(3) Derivative Formation*. The methyl esters were reacted with 15 ml of anhydrous methanol saturated with silver nitrate. The reaction was carried out for 20 hours at room temperature. The normal methyl esters and the reaction products from cyclopropenes were recovered from the reaction mixture by adding 30 ml of distilled water and by extracting twice with 10 ml of petroleum ether. The combined ether fractions were dried over anhydrous sodium sulfate and evaporated to a small volume in a stream of nitrogen. For the oils that contained a large amount of cyclopropenoid fatty acids (>5.0%), for example, the petroleum ether solution of methyl esters, the reaction products were directly injected into the gas-liquid chromatograph. In this experiment, baobab crude oil was considered to have a low level of CPFAs. Then, 1 *μ*l was injected into the GC under split mode of 60°C (Shimadzu GC-2010 equipped with an autosampler) with a capillary column, Supelco Carbowax size 30 m × 0.53 mm, injection temperature of 240°C, and detection temperature of 260°C under a flame ionization detector. The fatty acid methyl esters were identified by comparing retention with standard times and expressed as percentages of the total methyl esters.

#### 2.4.3. Effect of Heating on Fatty Acid Composition

The baobab crude oil from the three regions was subjected to three different high temperatures (150°C, 200°C, and 250°C) and boiling duration of 5, 10, 15, and 20 minutes in the microwave oven (800 watts). The unheated crude oil sample was used as control (corresponding to 0 min). In these 3 × 4 factorial experiments, temperature and time were the treatments. After the heating experiment, the quantification of fatty acid and CPFA composition was carried out as shown in Section 2.4.2. A total of 36 treatments and 9 control experiments were performed.

### 2.5. Statistical Analysis

Descriptive statistics were performed to quantify the amount of fatty acids (saturated and unsaturated fatty acids) and cyclic fatty acids (CPFAs) in baobab seed crude oil using the MS Excel spreadsheet. The samples were analysed in triplicate, and the data were presented as mean ± standard deviation (SD). One-way analysis of variance (ANOVA) was performed to test for significant differences between the composition of fatty acids and CPFAs in three different regions and treatments. ANOVA with Tukey's least significant difference (LSD) test was used in SPSS version 17.0 (SPSS, Chicago, IL, USA) to evaluate the differences between and within fatty acids and CPFAs. The differences were considered statistically significant at *p* < 0.05. A general linear model (GLM) ANCOVA in SPSS version 17.0 (IBM Corp., Chicago IL) was run using time as a fixed/random factor and temperature as a covariate to determine the effects of heat and boiling duration on the concentrations of fatty acids and CPFA composition.

## 3. Results

### 3.1. Physicochemical Properties of Baobab Seed Oil

The quality of crude baobab seed oil samples was determined by evaluating the physicochemical properties. The parameters analysed were free fatty acids (FFA), specific gravity, saponification value, unsaponifiable matter, peroxide value, and iodine value. Except for refractive index and unsaponifiable matter, no significant differences (*p* > 0.05) in the physicochemical properties of baobab crude oil samples were observed from the three selected semiarid regions of Tanzania (Table 2).

#### 3.1.1. Free Fatty Acids

The obtained results for the free fatty acids showed that the average free fatty acids for the baobab oil samples were 1.03 ± 0.05, 1.03 ± 0.05, and 1.06 ± 0.05 for Iringa, Dodoma, and Kilimanjaro, respectively (Table 2).

#### 3.1.2. Refractive Index

The results showed that the average refractive indexes at 26°C for the baobab crude oil were 1.05 ± 0.01, 1.07 ± 0.00, and 1.04 ± 0.00 for Iringa, Dodoma, and Kilimanjaro, respectively (Table 2).

#### 3.1.3. Specific Gravity of Baobab Crude Oil

The specific gravity value is a good measure of purity of oils. Fatty acids in the oil affect the specific gravity of oil; therefore, the higher the value in the chain length of fatty acid present in oil, the higher the rise in the specific gravity of oils. The average specific gravity of baobab crude oil samples at 25°C was 0.928 ± 0.001 in all the three sampled regions (Table 2).

#### 3.1.4. Saponification Value

Baobab crude oil samples showed higher saponification values of 219.87 ± 2.8, 217.45 ± 0.77, and 210.83 ± 0.45 mg/KOH for Iringa, Dodoma, and Kilimanjaro regions, respectively (Table 2). The highest average value was obtained from Iringa region, and the lowest value was obtained from Kilimanjaro region.

#### 3.1.5. Unsaponifiable Matter

Our study found that the average unsaponifiable matter values for baobab crude oil samples were 1.22 ± 0.06, 1.17 ± 0.09, and 0.90 ± 0.08 for Iringa, Dodoma, and Kilimanjaro regions, respectively (Table 2). The highest and the lowest values for baobab crude oil samples were obtained from Dodoma and Kilimanjaro regions, respectively.

#### 3.1.6. Peroxide and Iodine Values

The results showed that the average peroxide values of the baobab crude oil samples were 3.8 ± 0.61, 3.69 ± 0.10, and 3.83 ± 0.35 mEq/kg for Iringa, Dodoma, and Kilimanjaro regions, respectively. The highest values for baobab crude oil samples were obtained from Kilimanjaro, and the lowest values were obtained from Dodoma region. In this study, the average iodine values for baobab oil were 87.16 ± 5.3, 87.69 ± 0.61, and 90.70 ± 1.71 g/100 g for Iringa, Dodoma, and Kilimanjaro, respectively (Table 2).

### 3.2. Fatty Acid Composition in Baobab Seed Crude Oil

In this study, twelve fatty acids were identified and quantified from baobab seed oil collected from three different regions in the semiarid areas of Tanzania (Table 3). In our study, we found that the total composition of fatty acid in the baobab crude oil was 98.09 wt percent. The composition percentages of fatty acid in baobab crude oil were 68.59, 28.39, and 3.02 percent for unsaturated, saturated, and cyclic fatty acids, respectively. There were no significant differences (*p* > 0.05) in the compositions of saturated, unsaturated, and cyclic fatty acids between the three regions in the semiarid areas of Tanzania. However, there were significant differences (*F*_9,125_ = 236.252, *p* < 0.001) in the composition of the twelve fatty acids identified in baobab seed oil in the semiarid areas of Tanzania. Furthermore, there were significant differences (*F*_2,153_ = 11.335, *p* < 0.001) in the compositions of saturated, unsaturated, and cyclic acids within the region. The numbers of fatty acid identified included five saturated fatty acids, five unsaturated, and two cyclic fatty acids.

The saturated fatty acids identified in the crude oil were myristic, palmitic, palmitoleic, stearic, and arachidic. There were no significant differences (*p* > 0.05) in saturated fatty acid compositions in baobab seed oil. The highest and lowest composition quantity values of saturated fatty acids were found for baobab oil sample collected from Iringa and Dodoma regions, respectively. The average amount of saturated fatty acids was myristic 0.28-0.34%, palmitic 14.99-20.48%, palmitoleic 0.78-0.99%, stearic 1.15-2.16%, and arachidic 0.26-0.733%. The highest amount of palmitic fatty acid as opposed to other saturated fatty acids was detected in baobab seed oil. Furthermore, myristic acids were present in all the samples, although in low concentrations (<0.5%).

The unsaturated fatty acids identified were oleic, vaccenic, linoleic, linolenic, and arachidonic. Baobab seed oil was found to contain both mono (oleic and vaccenic) and polyunsaturated fatty acids (linoleic, linolenic, and arachidonic). Furthermore, there were no significant differences (*p* > 0.05) in unsaturated fatty acids in baobab seed oil. The most abundant fatty acids were palmitic, oleic, and linoleic acids (Table 3). The range of the amount of unsaturated fatty acid found in baobab oil was vaccenic 3.64-5.48%, oleic 24.4-29.52%, linoleic 21.20-24.86%, linolenic 12.14-26.69%, and arachidonic 0.54-1.30%. Furthermore, the study identified two cyclic fatty acids, namely, sterculic (0.97-1.45%) and dehydrosterculic (0.80-1.29%). There were significant differences (*F*_2,23_ = 13.30, *p* < 0.001) in the compositions of cyclic fatty acids in baobab seed oil.

### 3.3. Effect of Heating on Fatty Acid Compositions

Fatty acid (saturated, unsaturated, and cyclic fatty acids) results from the three regions were pooled together and considered as one sample since there were no observed significant differences in fatty acid composition. There was no significant main effect on fatty acid compositions (*p* = 0.125) when the samples were subjected to heating at a temperature of 150°C within 20 minutes. However, there was a significant main effect of temperature at 200°C on fatty acid compositions (*p* = 0.15). In addition, there was a significant main effect of temperature at 250°C on fatty acid compositions (*p* < 0.001) within 20 minutes.

#### 3.3.1. Effect of Heating on Saturated Fatty Acid Composition

**Figure 2:**
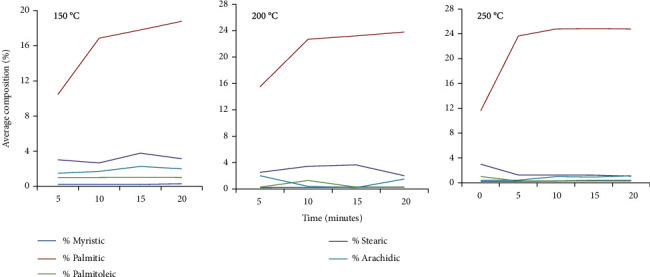
Percentage of saturated fatty acid compositions with a variation of time and temperature.

The results showed that heating temperature (range of the heating temperatures) had an influence on the fatty acid composition. There was a slight increase in temperature up to 200°C in the majority of the saturated fatty acids regardless of heating duration (Figure 2). It was interesting to observe that different saturated fatty acid compositions responded differently to changing temperatures. For example, a sharp increment of the percentage composition was observed during the first ten minutes of heating (at 150, 200, and 250°C) for palmitic acid and a slight increase was observed after 10 minutes of heating at different temperatures. However, myristic, stearic, and palmitoleic acid percentage compositions decreased with an increase in temperature (>250°C). All saturated fatty acid compositions decreased at a temperature of 250°C. There was a significant difference (*p* < 0.05) in palmitic acid composition in all the three temperatures. No significant difference (*p* > 0.05) was observed in the heating temperature and composition of myristic, palmitoleic, stearic, and arachidic (Figure 2).

In this study, heating temperatures had a marked effect on the fatty acid compositions. The saturated fatty acids such as palmitic, stearic, and arachidic showed an increase in composition at 150°C, while palmitoleic and myristic remained almost constant with changing heating temperatures. For the saturated fatty acids, compositions increased with an increase in temperatures. Interestingly, myristic, palmitic, palmitoleic, and arachidic increased with an increase in temperature (Figure 2).

#### 3.3.2. Effect of Heating on Unsaturated Fatty Acid Composition

**Figure 3:**
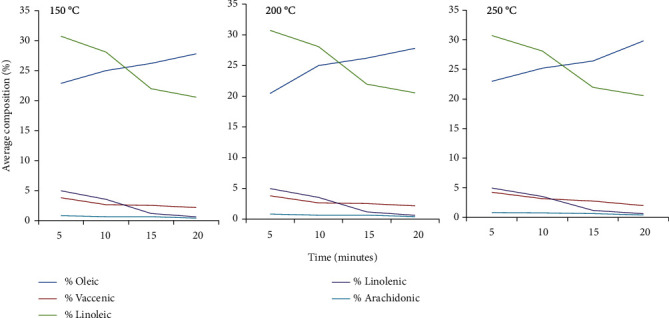
Percentage of unsaturated fatty acid composition with a variation of time and temperature.

At all the three temperatures (150°C, 200°C, and 250°C), the quantity of unsaturated fatty acids decreased as the temperature increased, probably due to degradation of polyunsaturated fatty acids. Remarkably, the quantity of oleic acid increased with an increase in temperature. The unsaturated fatty acids that decreased with an increase in temperature included vaccenic, linoleic, linolenic, and arachidonic acids (Figure 3). There were significant differences (*p* < 0.05) in linolenic acid composition at the three temperatures. Furthermore, there were significant differences (*p* < 0.05) in oleic fatty acid composition the three temperatures (150°C, 200°C, and 250°C). However, no significant differences (*p* > 0.05) were observed in the heating temperature and composition of vaccenic, linolenic, and arachidonic.

#### 3.3.3. Effect of Heating and Time on CPFA Composition

**Figure 4:**
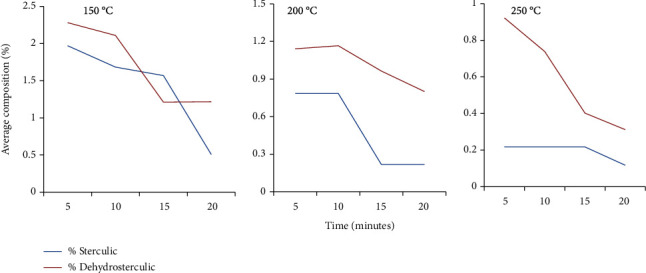
Percentage cyclopropenoid fatty acid composition with a variation of heating time and temperature.

The results showed that the CPFA compositions decreased with an increase in temperature (>150°C). The decomposition of all cyclic acids was relatively higher at the temperature of 200°C in 20 minutes (Figure 4). The decomposition decreased by about 2-8 percent and 5-15 percent of the original concentration in sterculic and dehydrosterculic fatty acids, respectively. There is a significant difference (*p* < 0.05) in sterculic and dehydrosterculic compositions at the three temperatures.

#### 3.3.4. Retention of Essential Fatty Acids during the CPFA Removal

At 250°C, the results showed that CPFA compositions decreased with time. Regarding essential fatty acids, oleic and palmitic acid increased, while linolenic decreased with heating time. Likewise, dehydrosterculic and sterculic acids decreased with heating time. After 15 minutes, the results showed that all CPFAs had reached the composition of below 0.4 percent, which was recommended by TFDA, while the essential fatty acids were retained (Figure 4).

## 4. Discussion

### 4.1. Physicochemical Properties of Baobab Crude Oil

The physicochemical properties from the three different regions, except for refractive index and unsaponifiable matter, did not show any significant differences (*p* > 0.05). This might be due to the fact that there were no variations in the environmental conditions in the regions where baobab fruits were collected (Table 2). The observed differences might be caused by the genotype of individual baobabs and the growth environments which affected the protein and subsequently changed the refractive index and unsaponifiable matter of the crude oil.

#### 4.1.1. Free Fatty Acids

The highest values of free fatty acids were obtained from Kilimanjaro region (Table 2). However, the values from all the three regions were lower than the values of 2.6 for baobab oil reported by Passera [39]. The low free fatty acids of baobab oil indicated that the oil may have a long shelf life and can be stored for a long time [39]. Baobab oil is extremely stable, and with proper storage conditions, it can have a highly variable shelf life of up to 5 years [39, 40]. Furthermore, the fatty acid profile could significantly change due to the storage and climatic conditions; the profile could increase with period of storage, air, heat, traces of metal, peroxides, light, or double bonds present in the oil and thus leading to the deterioration of the quality [41].

#### 4.1.2. Refractive Index

Our results for the baobab crude oil refractive index from the three regions were lower than the results reported by [41] which indicated baobab oil refractive index values of between 1.436 and 1.459 (Table 2). Our findings indicated slightly lower values than the values reported by [41] possibly because the oil solidified after exposure to air [42].

#### 4.1.3. Specific Gravity (SG)

The obtained average SG for baobab crude oil values (Table 2) from our study are within the WHO limit of between 0.91 and 0.93 [43]. The values are approximately similar to the values reported by Idris et al. [41] for the baobab oil. These values are within the range for vegetable oil such as the specific gravity ranges recommended by WHO/FAO for niger seed, sunflower, and palm oils of 0.917-0.92, 0.919-0.923, and 0.891-0.899, respectively [43]. The specific gravity indicates the purity of the oil. The lower the SG value the purer the oil and *vice versa*.

#### 4.1.4. Saponification Value

Saponification value determines the average chain length of the molecule and hence the estimated molecular weight of the fatty acid in the oil. Saponification equivalent is directly proportional to the average chain length of fatty acid present [44]. For example, the higher the saponification value, the lower the average molecular weight [45, 46]. The findings from our study (Table 2) are approximately similar to the findings in a study by Nkafamiya et al. [47] who recorded the saponification value of 196 ± 0.05 mgKOH/g for the baobab oil. The observed values are within the range of the other edible oils (187-196 mgKOH/g) and are used in soap making [48]. Ikhuoria and Maliki [49] observed that the saponification value of pepper fruit (*Dennettia tripetala*) oil was 159.33 mgKOH/g. Furthermore, a study by Nwinuka and Nwiloh [50] reported that the saponification value of the African pear oil was 143.76 mgKOH/g. Higher unsaponifiable matter results in restively lower saponification value which implies that the oil is suitable for soap making [49]. Baobab oil has been used in soap industries for many years in the world [49, 50].

#### 4.1.5. Unsaponifiable Matter

Unsaponifiable matter is that fraction of oils and fats which is not saponified by caustic alkali but is soluble in ordinary fat solvents. Unsaponifiable matters such as hydrocarbon, pigments, waxes, higher molecular weight alcohols, and sterols do not react with bases during the formation of soap. The average values obtained for the unsaponifiable matter from the three regions are similar to the value (1.7) reported by [41]. Also, Abubakar et al. [51] reported unsaponifiable matter value for baobab oil of 1.46. The low unsaponifiable matter indicates that the oil can be used for biodiesel production [41]. Baobab seed oil has been reported as one of the most suitable feedstock for biodiesel production, according to the fatty acid methyl ester profile that becomes one of the key factors [52]. Furthermore, low unsaponifiable matter of the baobab oil suggests that the oil could be edible because the observed value of unsaponifiable mater (Table 2) was within the recommended range of edible oils [51].

#### 4.1.6. Peroxide and Iodine Values

Peroxide value of oil measures the deterioration of oil over time. The higher the peroxide values, the lower the storage period of oil over time. The obtained average ranges for the baobab crude oil (Table 2) are within the ranges of 0-10 mEq/kg stipulated for freshly prepared vegetable oil [53]. Adebisi and Olagunju [54] demonstrated that peroxide values greater than 10 mEq/kg were highly susceptible to autooxidation when exposed to moisture or trace elements. The obtained values in our study were close to the value obtained by Babiker et al. [55] who reported the peroxide value for baobab oil of 4.08 mEq/kg. This suggests that the baobab oil has higher resistance to lipolytic hydrolysis and oxidation, and with proper storage conditions, it can be stored between 2 and 5 years without undergoing rancidity.

The iodine value has been used as a measure of the susceptibility of the oil to oxidation [56]. The iodine value shows the amount of double bonds present and the degree of unsaturation of the fatty acids in the specific oil. The obtained average iodine value implies a low percentage of unsaturated fatty acids in the baobab seed oil (Table 2). Our results are similar to the results in a study by Nkafamiya et al. [47] who reported the baobab oil iodine value (IV) of 87.9 ± 0.02 g/100 g, groundnut oil (84-99 g/100 g), olive (79-90 g/100 g), and castor oil (81-91 g/100 g). These ranges of iodine values suggest that the oil contains a low degree of unsaturation and can, therefore, be classified as nondrying edible oil because a range of 80-100 g/100 g iodine has been suggested for most edible oils [57].

### 4.2. Fatty Acid Composition in Baobab Crude Oil

The compositions of the fatty acids did not vary in the three different regions (Table 3). This implies that there were no variations in the environmental and soil conditions in the three regions. Conceivably, in the evolution of baobab populations into ecotypes with the possibility of genetic drift, the seed oil FA content pattern remained highly conserved. The results for the total fatty acid composition in baobab crude oil obtained in this study were similar to those reported by Idris et al. [41] who obtained 98.76 wt percent for the fatty acid composition. The compositions of palmitic, oleic, and linolenic fatty acids were higher compared to the compositions of other fatty acids. The higher fatty acid compositions of the baobab crude oil in this study were in the ranges of previous studies [9, 41]. It has been reported that as healthy fats, linoleic and oleic acids maintain cell membranes which provide energy and offer vitamin E which is a powerful antioxidant [32, 55]. Moreover, the oil contains CPFAs mainly sterculic and dehydrosterculic which is the characteristic property of Malvaceae family. Surprisingly, malvalic acid was below the detection limit in the baobab crude oil; hence, it deviates from the results reported in literature by [37]. The absence of malvalic acid could be attributed to the sample preparation method which involved oil extraction from baobab seeds using a pressing machine, leaving out the seed coat during fatty acid quantification [37]. The composition of the CPFAs found in the baobab crude oil sterculic (0.97-1.45%) and dehydrosterculic (0.80-1.29%) was higher than the recommended quantity for human consumptions [30].

### 4.3. Effect of Heating on Fatty Acid Composition

The variation in the heating temperatures influenced the compositions of saturated, unsaturated, and cyclic fatty acids. The observed effects of heating on fatty acid compositions were the same as those observed by Alil et al. [58]. The compositions of saturated fatty acids responded differently to the changing temperatures. During heating of the baobab oil, chemical reactions occurred. One among the reactions was that oxygen which reacted with unsaturated fatty acids resulted in hydroperoxide formation. A study by Ludger [59] observed that the geometrical isomerization of double bonds resulted in the formation of *trans* fatty acids during heating of oils. For the saturated fatty acids, the compositions increased with an increase in temperatures in our study (Figure 2).

Unsaturated fatty acid compositions decreased with an increase in temperatures probably due to the degradation of polyunsaturated fatty acids. A similar observation was made by Ali et al. [60] during thermo-oxidative degradation of canola oil. A decrease in unsaturated fatty acids was mainly due to the reaction of oxygen with unsaturated fatty acids resulting in hydroperoxides, which immediately degraded in further radical reactions at heating temperatures. Our results are similar to the results in a study by Marr et al. [61, 62] who reported a decrease in the proportions of unsaturated fatty acids as temperature decreased. In our study, it was evident that heating temperatures had a noticeable effect on the CPFA and fatty acid compositions. For example, heating reduced all CPFA composition significantly below 0.4 percent. This is the recommended level for human consumption by [30]. The major breakdown of CPFAs in the baobab oil occurred at 200°C and 250°C. In order to reduce the CPFAs in the baobab oil, heat is needed for postextraction treatments to make them fit for consumption [27]. These would be the best temperatures in the refining process of the baobab oil. Similar observations were made by [37]. Therefore, heat might be a good method of reducing the cyclopropenoid fatty acids from the baobab seed oils making them fit for consumption. However, further studies on the composition of fatty acids and the physicochemical parameters of baobab seed oil after the CPFA removal are necessary to determine the effect of heat on the quality of oil.

### 4.4. Equations

(1) $$\begin{array}{c} \text{Free fatty acid as oleic}=\frac{56.1\times V\times N}{W}, \end{array}$$

where *V* is the volume in ml of standard potassium hydroxide, *N* is the normality of the potassium hydroxide solution, and *W* is the weight in g of the sample

(2) $$\begin{array}{c} \text{Specific gravity at }\frac{30^{{}^{\circ}}\text{C}}{30^{{}^{\circ}}\text{C}}=\frac{C–B}{A–B}, \end{array}$$

where *A* is the weight in g of specific gravity bottle with oil at 30°C, *B* is the weight in g of specific gravity bottle at 30°C, and *C* is the weight in g of specific gravity bottle with water at 30°C.

(3) $$\begin{array}{c} \text{ Saponification value}=\frac{\left[56.1\;\left(B–S\right)N\right]}{W}, \end{array}$$

where *B* is the volume in ml of standard hydrochloric acid required for the blank, *S* is the volume in ml of standard hydrochloric acid required for the sample, *N* is the normality of the standard hydrochloric acid, and *W* is the weight in g of the oil/fat taken for the test.

(4) $$\begin{array}{c} \text{Unsaponifiable matter}=\frac{\left[100\left(A–B\right)\right]}{W}, \end{array}$$

where *A* is the weight in g of the residue, *B* is the weight in g of the free fatty acids in the extract, and *W* is the weight in g of the sample.

(5) $$\begin{array}{c} \text{Peroxide value}=\frac{\left[\left(A–B\right)*N*100\right]}{\text{Weight of the sample}}, \end{array}$$

where *A* is the ml of sodium thiosulphate used for blank, *B* is the ml of sodium thiosulphate used for sample, and *N* is the normality of sodium thiosulphate solution.

(6) $$\begin{array}{c} \text{Iodine value}=\frac{12.69\left(B–S\right)Xn}{W}, \end{array}$$

where *B* is the volume in ml of standard sodium thiosulphate solution required for the blank, *S* is the volume in ml of standard sodium thiosulphate solution required for the sample, *N* is the normality of the standard sodium thiosulphate solution, and *W* is the weight in g of the sample.

## 5. Conclusions

Our study identified 12 fatty acids, namely, myristic, palmitic, palmitoleic, stearic, arachidic, oleic, vaccenic, linoleic, linolenic, arachidonic, sterculic, and dehydrosterculic from the baobab oil. The presence of oleic and linoleic fatty acidy contributed to the medicinal value of the baobab oil. The physicochemical property values indicated that baobab oil has low unsaturation fatty acids making it edible as it is in liquid form at room temperature. The fatty acid profile of baobab seed oil is similar across all regions in the semiarid zone of Tanzania. Oleic, linoleic, and palmitic acids were found to be the major FAs, while stearic and linolenic acids were minor FAs. The possible existence of similar environmental conditions in the semiarid regions of Tanzania would not affect the FA pattern but might influence the quantities of some FAs of the seed oil. There is a need of assessing the pattern of FAs which are not included in this study in order to determine whether other FAs could be potential markers for baobab seed oil. The heating of baobab oil causes an increase of saturated fatty acids at temperatures of 150°C and 200°C. Sterculic and dehydrosterculic fatty acids were the CPFAs detected during this study. Further studies should be carried out to investigate the presence of malvalic acid in the baobab oil. It has been observed that a major breakdown of CPFA was at 200°C. The present results indicated that heating the baobab oil has an effect on the fatty acid composition as well as the amount of CPFAs. The temperature ranging from 200°C to 250°C of could be the optimal in the refining process of the baobab crude oil especially on the reduction of CPFA to the level that is recommended for human consumption.

## Figures and Tables

**Table 1:** Geographical location and environmental conditions of regions where baobab fruits were collected.

Region	Soil type (FAO/UNESCO classification)	Tree code	Latitude	Longitude	Altitude (m)	Temperature (°C)	Mean annual rainfall (mm)
Dodoma	Luvic Xerosol	DOM1	-5.124	35.771	708	28	600
		DOM2	-5.599	35.434	710	28	600
		DOM3	-5.941	36.260	1265	29	900
Iringa	Eutirc Cambisol	IR1	-7.557	35.213	793	29	600
		IR2	-7.416	35.573	512	29	900
		IR3	-7.434	35.754	583	29	900
Kilimanjaro	Luvic Xerosol	KL1	-3.554	37.618	640	29	600
		KL2	-4.254	38.123	985	26	800
		KL3	-3.912	37.618	1091	27	800

**Table 2:** Physicochemical properties of baobab seed oil.

Physiochemical properties	Region			
	Iringa	Dodoma	Kilimanjaro	*p* values
FFA (mEq/100 g oil)	1.03 ± 0.05	1.03 ± 0.05	1.06 ± 0.05	0.752
Specific gravity (g/ml oil)	0.928 + 0.001	0.928 + 0.001	0.928 + 0.001	0.47
Refractive index (at 26°C)	1.05 ± 0.01	1.07 ± 0.00	1.04 ± 0.00	0.03
Saponified matter (mEq NaOH/g of sample)	196.87 ± 2.8	196.45 ± 0.77	186.83 ± 0.45	0.114
Unsaponifiable matter (g/100 g)	1.22 ± 0.06	1.17 ± 0.09	0.90 ± 0.08	0.001
Peroxide value (mEq/kg)	3.8 ± 0.61	3.69 ± 0.10	3.83 ± 0.35	0.742
Iodine value (mEq iodine/g)	87.16 ± 5.31	87.69 ± 0.61	90.70 ± 1.71	0.407

**Table 3:** Fatty acid levels, expressed as % of total FAs in baobab seed oil.

Group	Fatty acid name	Chemical name (systematic name)	Abbreviation (bonds)	Average composition (%)		
				Dodoma	Iringa	Kilimanjaro
*Saturated fatty acids*	Myristic	C_14_H_28_O_2_	C14:0	0.28 ± 0.13	0.32 ± 0.14	0.34 ± 0.13
	Palmitic	C_16_H_32_O_2_	C16:0	18.38 ± 5.87	14.99 ± 6.87	20.48 ± 5.98
	Palmitoleic	C_16_H_30_O_2_	C16:1n-7	0.78 ± 0.38	0.85 ± 0.38	0.99 ± 0.39
	Stearic	C_17_H_35_CO_2_H	C18:0	1.341 ± 1.11	2.16 ± 1.6	1.15 ± 2.13
	Arachidic	C20H40O2	C20:0	1.18 ± 0.71	0.54 ± 0.70	1.3 ± 0.82
*Total*				**21.961**	**18.34**	**24.26**
*Unsaturated fatty acids*	Oleic	C_18_H_34_O_2_	18:1 cis-9	24.86 ± 3.97	21.20 ± 3.87	21.69 ± 3.97
	Vaccenic	C_18_H_34_O_2_	18:1 trans-11	2.81 ± 0.34	2.4 ± 0.24	2.52 ± 0.01
	Linoleic	C_18_H_32_O_2_	C18:2	3.92 ± 2.18	3.64 ± 1.18	5.48 ± 1.18
	Linolenic	C_18_H_30_O_2_	C18:3	21.69 ± 2.32	12.16 ± 1.34	26.14 ± 1.92
	Arachidonic	C_20_H_32_O_2_	C20:4	0.26 ± 0.20	0.733	0.53
*Total*				**59.47**	**40.133**	**56.36**
*Cyclic fatty acids*	Sterculic	C_19_H_34_O_2_		0.97 ± 0.56	1.45	1.17
	Dehydrosterculic	C_19_H_34_O_2_		1.19 ± 0.65	0.80	1.29
*Total*				**2.16**	**2.25**	**2.46**

## Data Availability

The data that support the findings of this study are available from the corresponding author upon reasonable request.
